# Defecation Patterns and Cardiovascular Outcomes in Acute Coronary Syndrome: The Influence of Stimulant Laxative Use

**DOI:** 10.1016/j.jacasi.2026.01.035

**Published:** 2026-05-16

**Authors:** Yasushi Matsuzawa, Kenichi Tsujita, Takaomi Kessoku, Masaomi Gohbara, Masanobu Ishii, Taishi Nakamura, Hisaya Kondo, Tomohiro Yoshii, Ryusuke Sekii, Jin Kirigaya, Kengo Terasaka, Hidefumi Nakahashi, Eiichi Akiyama, Masaaki Konishi, Toshihiro Yamada, Yuichiro Arima, Shinsuke Hanatani, Seiji Takashio, Hiroki Usuku, Yasuhiro Izumiya, Eiichiro Yamamoto, Masami Kosuge, Kazuo Kimura, Kiyoshi Hibi

**Affiliations:** a Department of Cardiovascular Medicine, Graduate School of Medical Sciences, Kumamoto University, Kumamoto, Japan; b Division of Cardiology, Yokohama City University Medical Center, Yokohama, Japan; c Department of Palliative Medicine and Gastroenterology, International University of Health and Welfare Narita Hospital, Narita, Japan; d Department of Gastroenterology, International University of Health and Welfare Graduate School of Medicine, Narita, Japan; e Department of Gastroenterology and Hepatology, Yokohama City University School of Medicine Graduate School of Medicine, Yokohama, Japan; f Department of Medical Information Science, Graduate School of Medical Sciences, Kumamoto University, Kumamoto, Japan; g Kawaguchi Cardiovascular and Respiratory Hospital, Saitama, Japan; h Department of Medical Science and Cardiorenal Medicine, Yokohama City University Graduate School of Medicine, Yokohama, Japan; i International Research Center for Medical Sciences (IRCMS), Kumamoto University, Kumamoto, Japan; j Department of Laboratory Medicine, Kumamoto University Hospital, Kumamoto, Japan; k Yokosuka City Hospital, Yokosuka, Japan

**Keywords:** acute coronary syndrome, cardiovascular events, defecation pattern, gut frailty, stimulant laxatives

## Abstract

**Background:**

Appropriate defecation patterns may be important for the prevention of recurrent cardiovascular events.

**Objectives:**

This study aimed to investigate the association of defecation patterns and laxative use during hospitalization with future cardiovascular events in patients with acute coronary syndrome (ACS).

**Methods:**

This 2-center retrospective observational study included 1,817 patients hospitalized for ACS. In addition to: 1) “average daily defecation frequency,” we comprehensively evaluated the following abnormal defecation patterns; 2) “% of nondefecation days;” 3) “consecutive nondefecation days;” and 4) “maximum daily defecation frequency.” Cutoff values were determined using maximization of the log-rank statistic. The primary outcome was a composite of all-cause mortality, myocardial infarction, ischemic stroke, and hemorrhagic stroke. Laxatives were categorized into stimulant and nonstimulant types, and their associations with defecation patterns and cardiovascular outcomes were assessed.

**Results:**

During a median follow-up of 48 months (IQR: 29-74 months), 375 of 1,817 patients (20.6 [95% CI: 18.8-22.6]%) developed the primary outcome. Frequent nondefecation days (adjusted HR for ≥33.8%: 1.549; 95% CI: 1.217-1.971; *P* < 0.001)—corresponding to at least 1 nondefecation day every 3 days—and high maximum daily defecation-frequency (adjusted HR for ≥5 times/d: 1.803; 95% CI: 1.273-2.554; *P* < 0.001) were significant predictors. Those with reduced defecation frequency more often used stimulant laxative, which was independently associated with the primary outcome (adjusted HR: 1.285; 95% CI: 1.003-1.646; *P* = 0.047).

**Conclusions:**

Abnormal defecation patterns and stimulant laxative use during ACS hospitalization were independent factors associated with an increased risk of future cardiovascular events.

Patients who survive acute coronary syndrome (ACS) are at a significantly elevated risk of recurrent cardiovascular events and overall mortality, despite the implementation of secondary prevention measures.^1^ For further improvement of the prognosis of ACS patients, it is necessary to explore residual risks and develop intervention methods for them.

The risk of cardiovascular events increases during straining in patients with constipation,^2^ but the gut may play a much more significant role in various aspects. Abnormal gut function may potentially contribute to cardiovascular diseases through mechanisms, including gut microbial dysbiosis, impairment of intestinal barrier function (commonly referred to as “Leaky gut syndrome”), systemic inflammation, autonomic nervous system dysregulation, and reduced secretion of cardioprotective incretins.^3^, ^4^, ^5^ Considering the various roles that the gut plays in the body, it is plausible that they influence both cardiovascular and noncardiovascular events in patients with ACS in the long term.

Previous studies have reported an association between chronic constipation and cardiovascular events.^6^, ^7^, ^8^ Physical frailty during hospitalization for acute myocardial infarction and subsequent hospital-acquired disability can have a long-term adverse impact on prognosis.^9^ Similar to the decline in physical function during hospitalization for acute illness, various abnormal patterns in defecation frequency during hospitalizations may also impact not only gut health and overall human health but also the prevention of recurrent cardiovascular events. Nevertheless, the relationship between defecation frequency during acute illness and subsequent adverse clinical outcomes remains unexplored. Additionally, the impact of laxative use on defecation patterns and cardiovascular outcomes has not been thoroughly investigated.

We hypothesized that abnormal patterns in defecation frequency, an indicator of “gut frailty,”^10^ are a residual risk factor in patients with ACS. Our primary aim was to examine the association of defecation frequency and laxative use during a general ward stay after intensive care with the occurrence of future major adverse cardiovascular events (MACE).

## Materials and Methods

### Study design and population

We conducted a retrospective, observational cohort study and enrolled consecutive patients with ACS who were admitted to Yokohama City University Medical Center and Kumamoto University Hospital, from January 1, 2012, to December 31, 2019. The inclusion criteria were age ≥18 years at enrollment, ACS diagnosis (unstable angina, non–ST-segment elevation myocardial infarction [NSTEMI], or ST-segment elevation myocardial infarction [STEMI]) according to the Japanese guideline.^11^ The exclusion criteria were in-hospital death and discharge from hospital with general ward stay ≤2 days, because it was difficult to evaluate defecation frequency. Patients who underwent coronary artery bypass grafting (CABG) for ACS as the index event were also excluded. CABG patients differ substantially from those undergoing percutaneous coronary intervention (PCI) or conservative treatment in terms of perioperative management, including surgical stress, prolonged immobilization, bowel preparation, and opioid use, all of which can strongly influence bowel habits. In addition, the prognostic factors and mechanisms of cardiovascular events in CABG patients are distinct from those in nonsurgical ACS, which could introduce significant heterogeneity in the analysis. Furthermore, in our cohort, laxatives were used in 92% of CABG patients, stimulant laxatives were used in 83% of cases, and general anesthesia was administered in all cases, making it difficult to accurately evaluate spontaneous defecation patterns in this subgroup. Clinical information was obtained from medical records.

This study was conducted in accordance with the guidelines of the Institutional Ethics Committees of 2 hospitals, which provided the ethical approvals (approval number 2766), and with the Declaration of Helsinki. Written informed consent was waived due to retrospective anonymized data collection. All patients had the opportunity to opt out through information posted on the webpages of both hospitals.

### Defecation patterns

Defecation data were collected from nursing records of individual patients. Defecation was primarily confirmed directly by nurses during routine care, and additional information was obtained from patient self-reports for bowel movements that occurred outside of nursing observation periods (eg, during the night). A defecation frequency of less than 3 times per week is commonly used to define chronic constipation. This criterion, however, cannot accurately assess an irregular defecation pattern, which includes both days with no defecation and days with frequent defecation. Thus, in addition to: 1) “average daily defecation frequency,” we comprehensively evaluated the following abnormal defecation patterns; 2) “% of nondefecation days”; 3) “consecutive nondefecation days”; and 4) “maximum daily defecation frequency.” Days spent in the intensive care unit or high-care unit were excluded from the calculation of defecation frequency, because bowel movements in the intensive care unit are frequently influenced by factors such as immobility, sedation, fasting, prolonged bed rest, mechanical ventilation, and mechanical circulatory device, which may not reflect patients’ usual bowel function. Because these factors are also related to both reduced bowel movement frequency and worse clinical outcomes, inclusion of intensive care unit data could have resulted in an overestimation of the association between defecation patterns and prognosis. The examples of assessments for the indicators of defecation frequency can be found in Supplemental Figure 1.

1) “Average daily defecation frequency” (times/day) = total number of defecations in a general ward/total days in a general ward.
2) “% of nondefecation days” (%) = (days without defecation/total days in a general ward) ∗100.
3) “Consecutive nondefecation days” (days) = the longest consecutive days without defecation.
4) “Maximum daily defecation frequency” (times) = the highest number of defecations in a single day.

### Definition of stimulant and nonstimulant laxatives

Stimulant laxatives were defined as those containing anthraquinone derivatives, diphenylmethane derivatives, castor oil, or herbal preparations containing rhubarb (Rhei Radix et Rhizoma). Nonstimulant laxatives were defined as other osmotic laxatives, epithelial function-modifying agents, and bile acid transporters.

### Antibiotic therapy

Because antibiotic administration can affect bowel movements, data on antibiotic use during hospitalization were collected. As many patients received only a single dose of antibiotics at the time of PCI, patients were additionally categorized according to the duration of antibiotic therapy as <3 days or ≥3 days.

### Follow-up and definitions of adverse events

All patients were followed up for mortality, myocardial infarction, stroke, and heart failure hospitalization by reviewing their medical records and conducting phone calls with them or their families between January and August 2023. We also evaluated cause-specific mortality (cardiovascular, noncardiovascular, and cancer mortality). Given that the gut frailty can have a wide-ranging impact on both cardiac and noncardiac conditions, we defined extended MACE as the primary outcome: a composite of the first occurrence of all-cause mortality, nonfatal myocardial infarction, nonfatal ischemic stroke, and nonfatal hemorrhagic stroke. Additionally, we defined 3-point MACEs (a composite of cardiovascular mortality, nonfatal myocardial infarction, and nonfatal ischemic stroke) as a secondary outcome to further evaluate the impact on purely cardiovascular events. We also analyzed each individual event as secondary outcome.

### Statistical analyses

Statistical analyses were conducted using JMP version 17.1.0 (SAS Institute Inc.) and R software version 4.5.2 (R Foundation for Statistical Computing). Variables are shown as mean ± SD, median (25th-75th percentile), or n(%). Group comparisons were made using Student’s *t*-tests for parametric data, Wilcoxon tests for nonparametric data, and Fisher exact test for categorical data. Spearman correlation analyzed relationships between indices. For Spearman rank correlation analyses, 95% CIs were estimated using Fisher z transformation. To assess a dose-response relationship between defecation frequency and cardiovascular event risk, data were divided into quartiles and risks compared. For time-to-event analyses, optimal cutoff values for each defecation frequency indicator were determined using maximization of the log-rank statistic. Prognostic analysis used the Kaplan-Meier method and log-rank test for significance. Univariate and multivariate Cox regression assessed independent prognostic factors, adjusting for age, sex, hypertension, diabetes, dyslipidemia, smoking, prior myocardial infarction, estimated glomerular filtration rate, STEMI, Killip class, PCI, intra-aortic balloon pumping, extracorporeal membrane oxygenation, food intake, and length of hospital stay. In the multivariate model examining the association between defecation patterns and clinical events, additional adjustments were made for the use of laxatives, the use of intestinal regulators, and the duration of antibiotic therapy. Multivariable analyses including 18 covariates (Model 1) were initially limited to the composite endpoint and all-cause mortality to prevent overfitting. Additional multivariable analyses were subsequently performed for other outcomes where feasible, using parsimonious models tailored to the number of events. Specifically, outcomes with a moderate number of events were analyzed using models adjusted for 10 clinically relevant covariates, whereas outcomes with fewer events were analyzed using models adjusted for 5 key covariates. The covariates included in each model are described in detail later in this article. Adjustment variables in Model 2 included age, sex, estimated glomerular filtration rate, STEMI, Killip class, intra-aortic balloon pumping, food intake, length of hospital stay, use of laxatives, and duration of antibiotic therapy. Adjustment variables in Model 3 included age, sex, Killip class, length of hospital stay, and use of laxatives. Missing data were assessed before multivariable analyses. The proportion of missing values for each variable included in the multivariable models was quantified, and missing data patterns were examined to evaluate overlap across covariates. The assumption of missing completely at random (MCAR) was formally assessed using Little’s MCAR test. The test rejected the null hypothesis (χ^2^ = 2271; df = 244; *P* < 0.001), indicating that missingness was not completely at random. However, pattern analysis demonstrated that missing data were largely confined to individual variables with minimal overlap across covariates. Given the limited degree of multivariable missingness and the sufficiently large number of complete cases, complete case analysis was applied for the primary multivariable regression models. Detailed results of the missing data assessment are provided in the Supplemental Materials (Supplemental Table 1, Supplemental Figure 2). The proportional hazards assumption was assessed using Schoenfeld residuals. For variables violating the proportional hazards assumption, time-dependent Cox regression models were applied by including interactions with log(time). Subgroup analyses were prespecified a priori based on factors known or hypothesized to influence prognosis and bowel function. These factors included demographic characteristics, comorbidities, disease severity, acute clinical presentation, and treatment-related variables. The predefined subgroups included age (<65 vs ≥65 years), sex, history of diabetes, history of myocardial infarction, chronic hemodialysis, diagnosis on admission (STEMI vs NSTEMI/unstable angina), presence of multivessel disease, Killip class (1 vs ≥2), left ventricular ejection fraction (>40% vs ≤40%), intra-aortic balloon pumping use, β-blocker use, calcium channel blocker use, laxative use, stimulant laxative use, duration of antibiotic therapy (<3 vs ≥3 days), length of hospital stay (<15 vs ≥15 days), and daily food intake (≥80% vs <80%). These subgroup analyses were conducted to assess the consistency of the associations between defecation-related variables and clinical outcomes across clinically relevant strata. Multiplicative interaction analyses were also prespecified a priori and were performed by including cross-product terms between the defecation-related variables and each subgroup variable in the multivariable regression models. A statistically significant interaction term was considered evidence of effect modification. ORs were estimated using univariate and multivariate logistic regression models to clarify the association between stimulant laxative use and abnormal defecation patterns. Multivariate logistic regression analyses were performed with each dichotomized defecation indicator (defined using prespecified cutoff values) as the dependent variable, and stimulant laxative use together with 16 clinically relevant covariates as independent variables. Laxative-related variables (laxatives and intestinal regulators) were excluded from the covariate set to avoid overadjustment. Two-sided tests were used, with significance set at *P* < 0.05.

## Results

**Figure 1:**
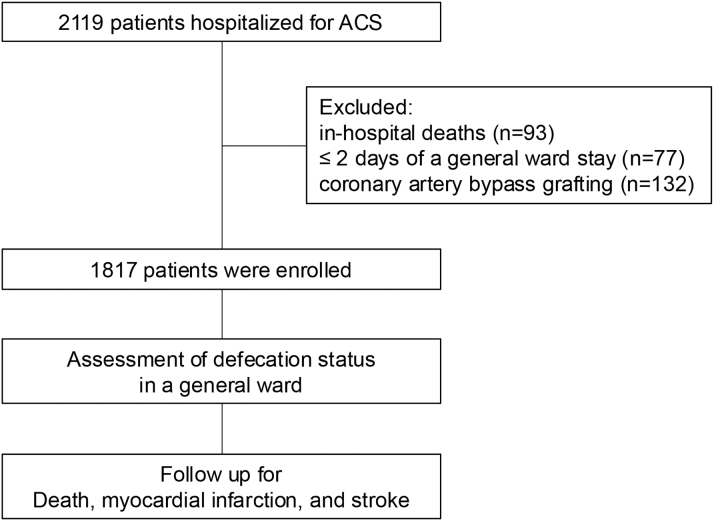
Study Population Flowchart This flowchart depicts the selection of the study cohort. Among 2,119 patients hospitalized for ACS, we excluded those who died during hospitalization (n = 93), those discharged from the general ward within 2 days (n = 77), and those who underwent CABG (n = 132). A total of 1,817 patients were included for assessment of in-hospital defecation status and followed longitudinally for cardiovascular outcomes, including all-cause death, myocardial infarction, and stroke. ACS = acute coronary syndrome; CABG = coronary artery bypass grafting.

In total, 2,119 patients were initially enrolled. Figure 1 shows the patient enrollment process. After excluding patients with in-hospital deaths (n = 93), those with ≤2 days of general ward stay (n = 77), and those with CABG (n = 132), 1,817 patients remained (sex: 80.2% male; mean age: 68 years). The median (IQR) length of stay in general wards was 12 (8-16) days, and the median (IQR) values for the 4 defecation indicators were 0.80 (0.57-1.08) times/d for the “average daily defecation frequency,” 33.3 (17.6-50.0)% for the “% of nondefecation days,” 2 (1-3) days for the “consecutive nondefecation days,” and 2 (1-3) times for the “maximum daily defecation frequency” (Supplemental Figure 3). The cutoff values of 4 indicators of defecation for predicting the primary outcomes were 0.19 for the “average daily defecation frequency,” 33.8% for the “% of nondefecation days,” 6 days for the “consecutive nondefecation days,” and 5 times for the “maximum daily defecation frequency.”

### Clinical events during the follow-up

During the follow-up period (median: 48 months; IQR: 29-74 months), 375 of 1,817 (20.6%) patients developed the primary outcome. In total, 216 of 1,817 patients (11.9% [95% CI: 10.5%-13.5%]) died, including 90 cardiovascular deaths (5.0% [95% CI: 4.0%-6.0%]) and 126 noncardiovascular deaths (6.9% [95% CI: 5.9-8.2%]). Overall, 256 of 1,817 patients (14.1% [95% CI: 12.6%-15.8%]) experienced 3-point MACE. Among these, 135 patients (7.4% [95% CI: 6.3%-8.7%]) had nonfatal myocardial infarction, 45 (2.5% [95% CI: 1.9%-3.3%]) had nonfatal ischemic stroke, 23 (1.3% [95% CI: 1.0%-1.9%]) had nonfatal hemorrhagic stroke, and 104 (5.7% [95% CI: 4.7%-6.9%]) were hospitalized for heart failure.

### Missing data

There are missing data regarding multivessel disease in 184 patients, left ventricular ejection fraction in 312, estimated glomerular filtration rate in 283, 153 in PCI, intra-aortic balloon pumping in 2, and daily food intake in 20. These patients were excluded from the multivariate analysis, which includes 18 variables, resulting in the number of patients in the multivariate models of 1,364, among whom the primary outcome occurred in 287 patients. Among the study population, 1,364 patients had complete data for all variables included in the multivariable models and were included in the primary analyses. The proportions of missing data for each variable are summarized in Supplemental Table 1. Pattern analysis showed that missing data were largely confined to individual variables without systematic overlap across covariates (Supplemental Figure 2). Little’s MCAR test rejected the assumption of missing completely at random (chi-square = 2,271; df = 244; *P* <0.001).

### Defecation frequency and the events

The univariate Cox hazard analysis revealed that “average daily defecation frequency” was not significantly associated with any event (Table 1). However, “% of nondefecation days” was associated with the primary outcome, 3-point MACE, all-cause death, noncardiovascular death, and nonfatal myocardial infarction. “Consecutive nondefecation days” was associated with the primary outcome, all-cause death, noncardiovascular death, and nonfatal ischemic stroke, and “maximum daily defecation frequency” was associated with the primary outcome, all-cause death, noncardiovascular death, cancer death, and heart failure hospitalization.

**Table 1:** HRs for Adverse Event Risk According to Defecation Patterns

	No. of Events (% [95% CI])	Univariate		*P* Value	Multivariate		
		HR	95% CI		HR	95% CI	*P* Value
		per 0.1 decrease in average daily defecation frequency					
The primary outcome: the extended MACE	375 (20.6 [18.8-22.6])	0.999	0.981-1.020	0.988	1.006	0.983-1.030	0.598
3-point MACE	256 (14.1 [12.6-15.8])	1.011	0.987-1.038	0.367	1.012	0.984-1.042	0.397
All-cause death	216 (11.9 [10.5-13.5])	0.988	0.967-1.012	0.325	0.993	0.966-1.021	0.623
Cardiovascular death	90 (5.0 [4.0-6.0])	1.014	0.974-1.060	0.514	0.999	0.957-1.044	0.980
Noncardiovascular death	126 (6.9 [5.9-8.2])	0.974	0.951-1.004	0.083	0.980	0.953-1.015	0.213
Cancer death	59 (3.2 [2.5-4.2])	0.977	0.944-1.022	0.289	0.983	0.942-1.026	0.431
Nonfatal myocardial infarction	135 (7.4 [6.3-8.7])	1.023	0.988-1.062	0.214	1.027	0.989-1.070	0.199
Nonfatal ischemic stroke	45 (2.5 [1.9-3.3])	1.008	0.958-1.073	0.795	1.012	0.961-1.079	0.685
Nonfatal hemorrhagic stroke	23 (1.3 [1.0-1.9])	0.991	0.936-1.079	0.824	0.984	0.912-1.062	0.675
Heart failure hospitalization	104 (5.7 [4.7-6.9])	0.991	0.961-1.028	0.596	1.008	0.968-1.050	0.701
		per 10% increase in % of nondefecation days					
The primary outcome: the extended MACE	375 (20.6 [18.8-22.6])	1.093	1.051-1.137	<0.001	1.099	1.048-1.151	<0.001
3-point MACE	256 (14.1 [12.6-15.8])	1.086	1.035-1.139	<0.001	1.101	1.042-1.164	<0.001
All-cause death	216 (11.9 [10.5-13.5])	1.087	1.032-1.145	0.002	1.068	1.001-1.139	0.046
Cardiovascular death	90 (5.0 [4.0-6.0])	1.080	0.995-1.169	0.067	1.037	0.942-1.142	0.459
Noncardiovascular death	126 (6.9 [5.9-8.2])	1.093	1.020-1.168	0.012	1.077	0.991-1.167	0.075
Cancer death	59 (3.2 [2.5-4.2])	1.017	0.914-1.126	0.751	1.030	0.923-1.142	0.591
Nonfatal myocardial infarction	135 (7.4 [6.3-8.7])	1.120	1.048-1.193	<0.001	1.121	1.043-1.203	0.002
Nonfatal ischemic stroke	45 (2.5 [1.9-3.3])	1.108	0.988-1.238	0.079	1.115	0.995-1.249	0.061
Nonfatal hemorrhagic stroke	23 (1.3 [1.0-1.9])	1.100	0.934-1.282	0.246	1.088	0.923-1.269	0.297
Heart failure hospitalization	104 (5.7 [4.7-6.9])	1.010	0.973-1.041	0.596	1.026	0.936-1.124	0.585
		per 1 day increase in consecutive nondefecation days					
The primary outcome: the extended MACE	375 (20.6 [18.8-22.6])	1.052	1.002-1.102	0.041	1.036	0.976-1.099	0.243
3-point MACE	256 (14.1 [12.6-15.8])	1.010	0.947-1.071	0.764	0.987	0.915-1.064	0.726
All-cause death	216 (11.9 [10.5-13.5])	1.110	1.047-1.172	<0.001	1.079	1.000-1.159	0.051
Cardiovascular death	90 (5.0 [4.0-6.0])	1.097	0.999-1.194	0.052	1.027	0.915-1.151	0.653
Noncardiovascular death	126 (6.9 [5.9-8.2])	1.119	1.035-1.200	0.005	1.145	1.043-1.256	0.005
Cancer death	59 (3.2 [2.5-4.2])	1.118	0.995-1.238	0.060	1.099	0.976-1.239	0.120
Nonfatal myocardial infarction	135 (7.4 [6.3-8.7])	0.924	0.833-1.015	0.104	0.948	0.846-1.053	0.341
Nonfatal ischemic stroke	45 (2.5 [1.9-3.3])	1.141	1.004-1.274	0.044	1.122	0.986-1.276	0.081
Nonfatal hemorrhagic stroke	23 (1.3 [1.0-1.9])	0.800	0.581-1.035	0.096	0.814	0.587-1.070	0.180
Heart failure hospitalization	104 (5.7 [4.7-6.9])	1.044	0.947-1.139	0.375	0.958	0.862-1.064	0.426
		per 1 time increase in maximum daily defecations frequency					
The primary outcome: the extended MACE	375 (20.6 [18.8-22.6])	1.081	1.042-1.116	<0.001	1.055	1.009-1.104	0.020
3-point MACE	256 (14.1 [12.6-15.8])	1.027	0.970-1.079	0.340	1.011	0.952-1.075	0.713
All-cause death	216 (11.9 [10.5-13.5])	1.126	1.081-1.166	<0.001	1.100	1.044-1.160	<0.001
Cardiovascular death	90 (5.0 [4.0-6.0])	1.061	0.971-1.136	0.172	1.037	0.947-1.136	0.428
Noncardiovascular death	126 (6.9 [5.9-8.2])	1.157	1.105-1.203	<0.001	1.164	1.093-1.238	<0.001
Cancer death	59 (3.2 [2.5-4.2])	1.159	1.082-1.224	<0.001	1.137	1.049-1.221	<0.001
Nonfatal myocardial infarction	135 (7.4 [6.3-8.7])	0.974	0.883-1.057	0.561	0.984	0.889-1.089	0.753
Nonfatal ischemic stroke	45 (2.5 [1.9-3.3])	1.072	0.953-1.163	0.217	1.051	0.924-1.156	0.370
Nonfatal hemorrhagic stroke	23 (1.3 [1.0-1.9])	1.038	0.838-1.188	0.685	1.085	0.900-1.310	0.394
Heart failure hospitalization	104 (5.7 [4.7-6.9])	1.080	1.005-1.144	0.037	0.995	0.911-1.085	0.904

Values are n (%).

The primary outcome was the extended MACE, which consisted of all-cause death, myocardial infarction, and ischemic and hemorrhagic stroke. The 3-point MACE consisted of cardiovascular death, myocardial infarction, and ischemic stroke. To minimize overfitting, the number of covariates included in the multivariable models was tailored to the number of events for each outcome. Model 1 was applied to the composite primary endpoint and all-cause mortality and included 18 covariates: age, sex, hypertension, diabetes, dyslipidemia, smoking status, prior myocardial infarction, estimated glomerular filtration rate, STEMI, Killip class, PCI, intra-aortic balloon pumping, extracorporeal membrane oxygenation, food intake, length of hospital stay, use of laxatives, use of intestinal regulators, and duration of antibiotic therapy. Model 2 was applied to outcomes with a moderate number of events (cardiovascular death, noncardiovascular death, nonfatal myocardial infarction, and heart failure hospitalization) and included 10 covariates: age, sex, estimated glomerular filtration rate, STEMI, Killip class, intra-aortic balloon pumping, food intake, length of hospital stay, use of laxatives, and duration of antibiotic therapy. Model 3 was applied to outcomes with fewer events (cancer death, nonfatal ischemic stroke, and nonfatal hemorrhagic stroke) and included 5 covariates: age, sex, Killip class, length of hospital stay, and use of laxatives.

MACE = major adverse cardiovascular event; PCI = percutaneous coronary intervention; STEMI = ST-segment elevation myocardial infarction.

**Figure 2:**
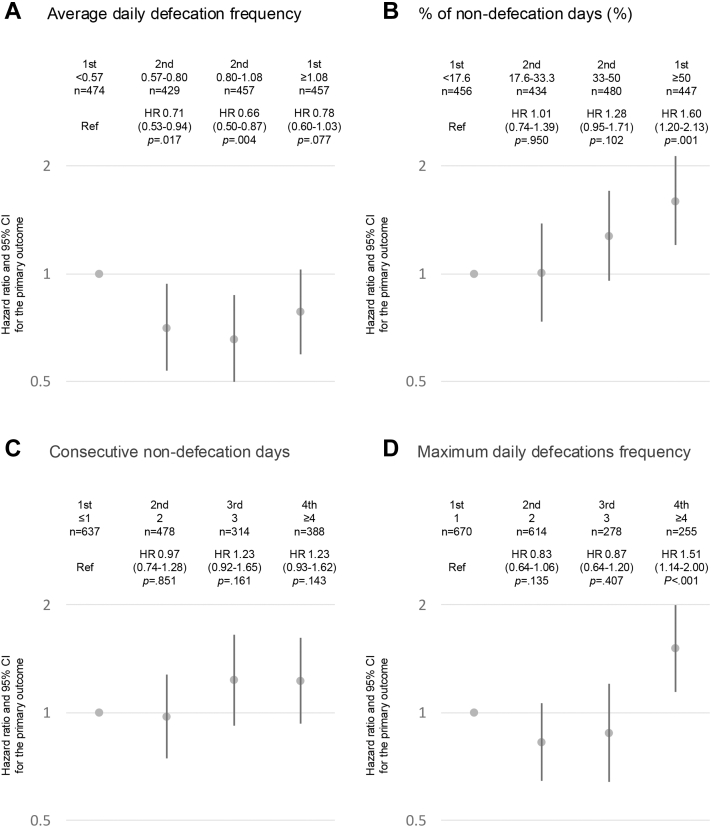
Defecation Frequency and Risk of Primary Outcome This figure shows HRs for the primary outcome according to quartiles of 4 indicators of in-hospital defecation frequency: (A) average daily defecation frequency, (B) percentage of nondefecation days, (C) consecutive nondefecation days, and (D) maximum daily defecation frequency. HRs were estimated using univariate Cox proportional hazards models. Higher proportions of nondefecation days and higher maximum daily defecation frequency were associated with increased long-term cardiovascular risk.

**Figure 3:**
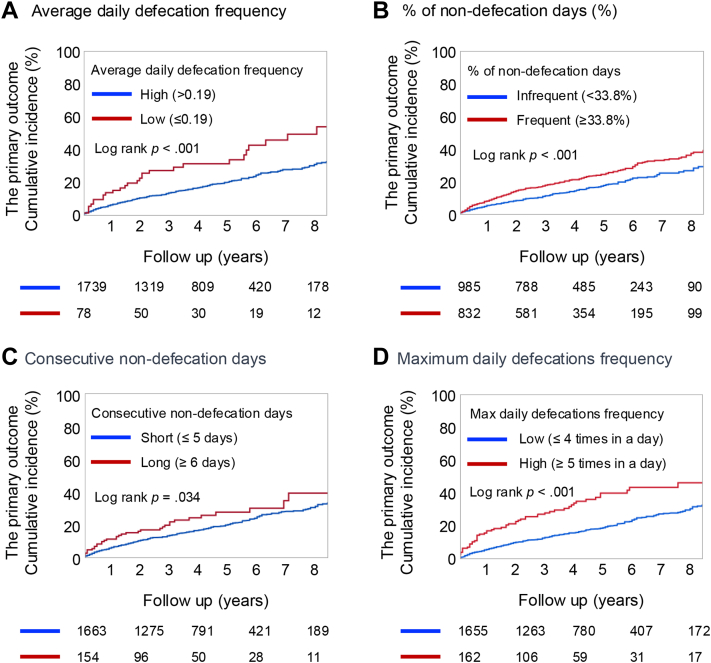
Primary Outcome by Defecation Indicators This figure presents Kaplan-Meier curves for the composite primary outcome according to 4 in-hospital defecation indicators: (A) average daily defecation frequency, (B) percentage of nondefecation days, (C) consecutive nondefecation days, and (D) maximum daily defecation frequency. Groups were categorized using data-driven cutoff values (0.19, 33.8%, ≥6 days, and ≥5 times/d, respectively).

Figure 2 illustrates the relationship between each indicator quartile and the primary outcome. From this graph, it can be inferred that the relationship between the 4 defecation indicators and the primary outcome risk is not linear. Therefore, we categorized the patients into 2 groups based on cutoff values. Figure 3 shows the univariate cumulative event rate analyses (Kaplan-Meier) and log-rank tests using the cutoff values of the 4 defecation indicators, illustrating their association with the primary outcome. The results of Cox hazard analyses comparing 2 groups based on cutoff values were shown in Table 2. In patients with low “average daily defecation frequency” (adjusted HR: 2.199; 95% CI: 1.399-3.455; *P* < 0.001), those with frequent nondefecation days (≥33.8%; adjusted HR: 1.549; 95% CI: 1.217-1.971; *P* < 0.001), and those with high maximum daily defecation frequency (≥5/d; adjusted HR: 1.803; 95% CI: 1.273-2.554; *P* < 0.001), the risk of the primary outcome is significantly higher than in comparators. The proportional hazards assumption was satisfied for “average daily defecation frequency” (*P* = 0.823), “frequency of nondefecation days” (*P* = 0.823), and “consecutive nondefecation days” (*P* = 0.172) (Supplemental Figure 4). In contrast, a significant violation of the proportional hazards assumption was observed for “maximum daily defecation frequency” (*P* = 0.004). Schoenfeld residual plots demonstrated a clear time-dependent pattern, indicating that the HR for this variable changed over time. In time-dependent Cox regression analysis, a maximum daily defecation frequency ≥5 times was associated with a markedly increased risk of the composite outcome in the early phase of follow-up (HR: 3.675; 95% CI: 2.307-5.855; *P* < 0.0001), although this effect significantly attenuated over time (time-dependent HR per log time: 0.794; 95% CI: 0.681-0.926; *P* = 0.003). These findings indicate that the prognostic impact of maximum daily defecation frequency is time dependent rather than constant over the follow-up period.

**Table 2:** Comparison of the Primary Outcome Risk Between 2 Groups Based on the Defecation Indicators

No. of Patients With the Primary Outcome/Total No. of Patients (%)		Log Rank *P* Value	Crude HR (95% CI)	*P* Value	Adjusted HR (95% CI)	*P* Value
Average daily defecation frequency						
>0.19	≤0.19		≤0.19 vs >0.19			
345/1,739 (19.8%)	30/78 (38.5%)	<0.001	2.093 (1.440-3.040)	<0.001	2.199 (1.399-3.455)	<0.001
% of nondefecation d						
<33.8%	≥33.8%		≥33.8% vs <33.8%			
168/985 (17.1%)	207/832 (24.9%)	<0.001	1.542 (1.258-1.891)	<0.001	1.549 (1.217-1.971)	<0.001
Consecutive nondefecation d						
≤5 d	≥6 d		≥6 d vs ≤5 d			
339/1,663 (20.4%)	36/154 (23.4%)	0.034	1.446 (1.025-2.040)	0.036	1.278 (0.847-1.929)	0.242
Maximum daily defecations frequency (n)						
≤4/d	≥5/d		≥5/d vs ≤4/d			
319/1,655 (19.3%)	56/162 (34.6%)	<0.001	2.022 (1.522-2.688)	<0.001	1.803 (1.273-2.554)	<0.001

Values are n (%).

The primary outcome was the extended MACE, which consisted of all-cause death, myocardial infarction, and ischemic and hemorrhagic stroke.

Abbreviations as in Table 1.

**Figure 4:**
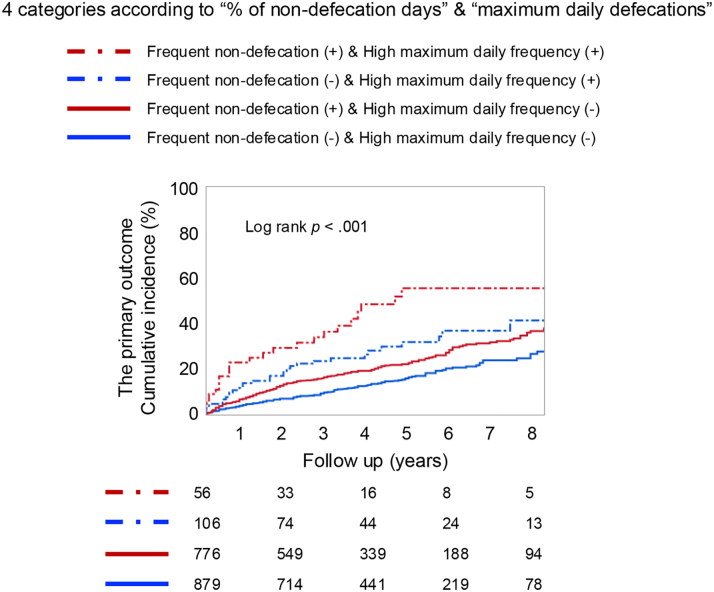
Primary Outcome by Combined Defecation Categories This figure illustrates Kaplan-Meier curves for the composite primary outcome stratified by 4 combined categories based on 2 defecation indicators: the percentage of nondefecation days (cutoff: 33.8%) and maximum daily defecation frequency (cutoff: ≥5 times/d). Patients with both frequent nondefecation days and high maximum defecation frequency exhibited the highest cumulative incidence of cardiovascular events. The curves demonstrate the additive prognostic impact of these defecation patterns during long-term follow-up.

Because “average daily defecation frequency” can be offset by days without defecation and days with frequent defecation, we performed additional analyses using 2 components, “% of nondefecation days” and “maximum daily defecation frequency.” When patients are divided into 4 groups using the cutoff values for “% of nondefecation days" and “maximum daily defecations frequency," a defecation pattern characterized by infrequent defecations on certain days but high-frequency defecations on other days was prognostically unfavorable (Figure 4).

### Patients’ characteristics

The baseline patients’ demographics and clinical characteristics according to the “% of nondefecation days” and the “maximum daily defecations frequency” are listed in Supplemental Tables 2 and 3. The group with frequent nondefecation days had fewer cases of STEMI compared with the group with infrequent nondefecation days. Additionally, the former group had a higher prevalence of prior myocardial infarction, more dialysis patients, lower food intake, and shorter hospital stays. There was a modest negative correlation between the “% of nondefecation days” and the “maximum daily defecations frequency” (Spearman ρ: −0.346; 95% CI: −0.385 to −0.304; *P* < 0.001). In the frequent nondefecation days group (defined as ≥33.8% nondefecation days), the prevalence of high “maximum daily defecation frequency” (≥5 times per day) was significantly lower (*P* = 0.003) (Supplemental Table 2). This suggests that these 2 indicators reflect distinct defecation pattern abnormalities.

The high maximum daily defecations frequency group was characterized by older age, poorer kidney and heart function, a higher proportion of severe cases requiring intra-aortic balloon pumping or venoarterial extracorporeal membrane oxygenation, and more frequent use of antibiotics compared with the low “maximum daily defecations frequency” group (Supplemental Table 3).

### Subgroup analysis

The subgroup analyses are shown in Supplemental Figures 5 and 6. The higher risks in the frequent nondefecation days group and the high maximum daily defecations frequency group for the primary outcome seemed to be consistent across all the subgroups.

### The association of laxative use with defecation abnormalities and clinical events

**Figure 5:**
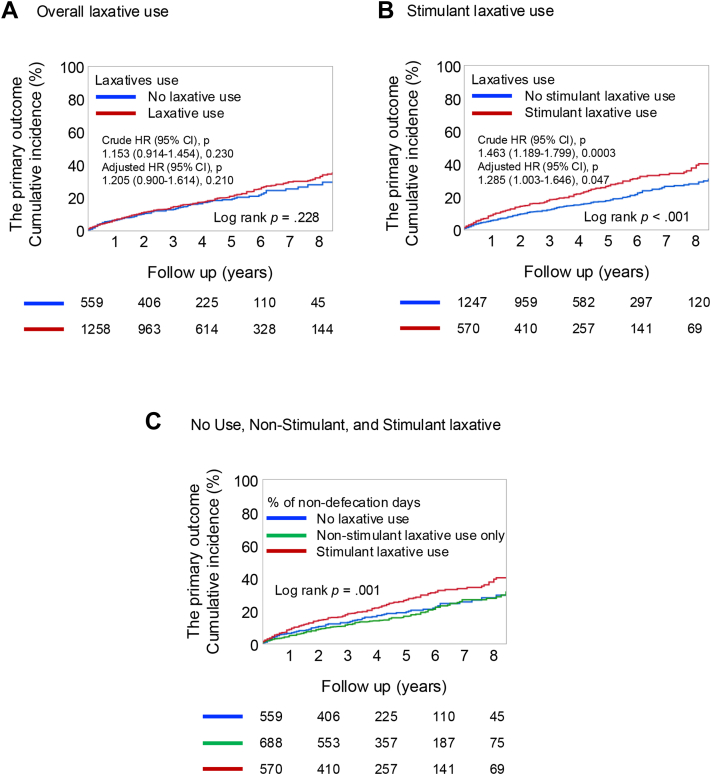
Primary Outcome by Laxative Use Categories This figure presents Kaplan-Meier curves evaluating the association between laxative use and the composite primary outcome. (A) Comparison of patients with overall laxative use vs no laxative use. (B) This focuses specifically on stimulant laxative use, demonstrating a higher cumulative incidence of cardiovascular events among stimulant users. (C) This further stratifies patients into 3 categories—no laxative use, nonstimulant laxative use only, and stimulant laxative use. HRs and CIs from Cox models are displayed within each panel.

In patients with a high frequency of nondefecation days (≥33.8%), stimulant laxatives were used more frequently (359 of 832 patients [43.2%]) than in those with a low frequency of nondefecation days (<33.8%; 211 of 985 patients [21.4%]; *P* < 0.001) (Supplemental Table 2). Similarly, stimulant laxative use was more frequent among patients with a high maximum daily defecation frequency (≥5 times/d; 79 of 162 patients [48.8%]) compared with those with a lower maximum daily defecation frequency (≤4 times/d; 491 of 1,655 patients [29.7%]; *P* < 0.001) (Supplemental Table 3). In multivariate logistic regression analyses, a high frequency of nondefecation days (≥33.8%) remained independently associated with stimulant laxative use after adjustment for other covariates (Table 3). A total of 570 of 1,817 patients (31.4%) received stimulant laxatives during hospitalization, and, among users, the median (IQR) frequency of administration was 0.12 (0.07-0.22) times/d. Most stimulant laxatives used in this study were senna-based agents. Patients were further classified into 3 groups according to stimulant laxative use frequency (none, low, and high). Using the no-use group as the reference, the HR (95% CI) for cardiovascular events was 1.524 (1.184-1.961; *P* = 0.001) in the low-frequency group and 1.394 (1.061-1.831; *P* = 0.017) in the high-frequency group, with no significant difference between the 2 laxative-use groups. Figure 5 presents Kaplan-Meier curves and Cox hazard analyses examining the association between laxative use and cardiovascular outcomes. Although overall laxative use was not associated with cardiovascular outcomes (Figure 5A), patients using stimulant laxatives had a higher risk of cardiovascular events (Figure 5B and 5C). This association remained significant in the multivariable Cox hazard analysis (HR: 1.285; 95% CI: 1.003-1.646; *P* = 0.047).

**Table 3:** Association Between Stimulant Laxative Use and Various Defecation Abnormality Indicators

**Defecation Abnormality Indicator**	**Stimulant Laxative Use**		**Stimulant Laxative User vs Non-user**					
	**(−)** **(n = 1,247)**	**(+)** **(n = 570)**						
	**% of Patients With Defecation Abnormality**		**Crude OR for Abnormal Defecation**	**95% CI**	***P* Value**	**Adjusted OR for Abnormal Defecation**	**95% CI**	***P* Value**
Average daily defecation frequency ≤0.19	3.4	6.3	1.934	1.225-3.054	0.005	3.338	1.870-5.957	<0.001
% of nondefecation days ≥33.8%	37.9	63.0	2.784	2.268-3.417	<0.001	3.149	2.448-4.050	<0.001
Consecutive nondefecation days ≥6 d	4.9	16.3	3.791	2.698-5.326	<0.001	2.637	1.666-4.174	<0.001
Maximum daily defecations frequency ≥5/d	6.7	13.9	2.256	1.629-3.125	<0.001	1.245	0.800-1.938	0.332

Values are n (%).

Abbreviations as in Table 1.

## Discussion

“Frequent nondefecation days” (≥33.8%) and high “maximum daily defecations frequency” (≥5 times/d) were independently associated with a heightened risk of future cardiovascular events. Although the cutoff values were determined using maximization of the log-rank statistic, the threshold for “frequent nondefecation days” (≥33.8%) indicates that patients had no bowel movement on more than one-third of the days—equivalent to at least 1 nondefecation day every 3 days. Patients with reduced defecation frequency were more likely to use stimulant laxatives; however, stimulant laxative use was associated with high “maximum daily defecation frequency” (≥5 times/d) and was also an independent predictor of cardiovascular events. These key findings are summarized schematically in the Central Illustration. These findings suggest that maintaining regular defecation may be important for favorable clinical outcomes.

**Central Illustration:**
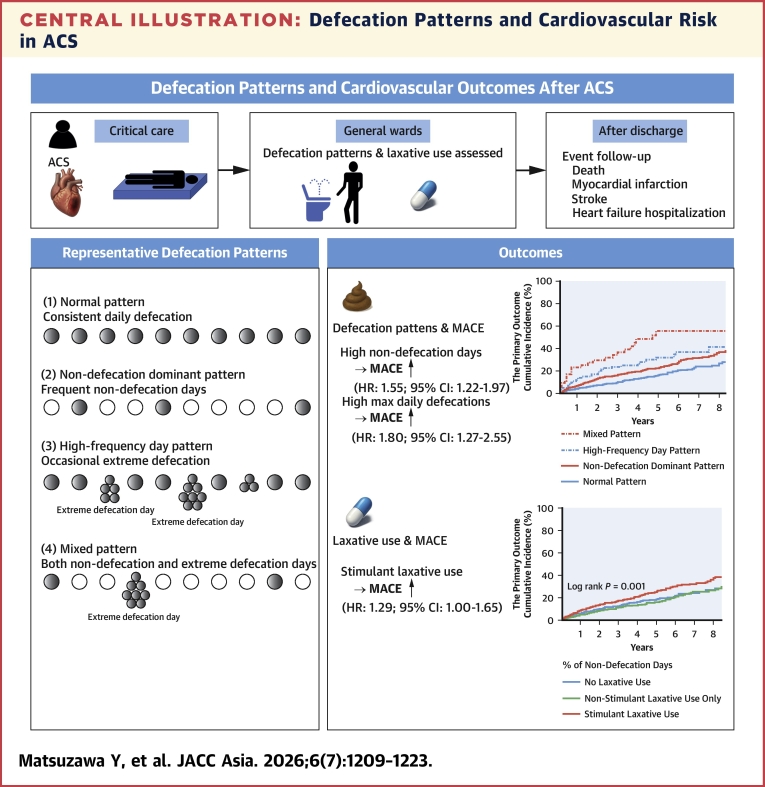
Defecation Patterns and Cardiovascular Risk in ACS This central illustration summarizes how 2 abnormal in-hospital defecation patterns—frequent nondefecation days and high maximum daily defecation frequency—were assessed in patients admitted with ACS. Representative clinical examples demonstrate how these patterns were classified. Kaplan-Meier curves show that patients exhibiting either or both patterns had significantly higher rates of MACE during long-term follow-up. Additional analyses indicate that stimulant laxative use was also associated with increased cardiovascular risk. These findings highlight the clinical relevance of routine monitoring of bowel habits and laxative use for risk stratification in ACS care. ACS = acute coronary syndrome; MACE = major adverse cardiovascular events.

The observed association between frequent in-hospital nondefecation days and subsequent cardiovascular events in patients with ACS in this study aligns with prior research findings indicating that “chronic constipation is linked to an adverse cardiovascular prognosis.”^6^, ^7^, ^8^ Notably, the cutoff value used in this study (≥33.8% nondefecation days) corresponds to having at least 1 nondefecation day every 3 days, which is consistent with the commonly accepted clinical definition of constipation (fewer than 3 bowel movements per week). This alignment supports the clinical relevance of our findings and suggests that even during short-term hospitalization, defecation frequency may serve as a meaningful indicator of future cardiovascular risk. The frequent nondefecation days group had a higher proportion of patients who had previously experienced myocardial infarction or developed renal failure requiring chronic dialysis. By including ACS events at the inclusion of this study, it can be inferred that patients with impaired gut function had a higher frequency of recurrent cardiovascular events. Furthermore, the frequent nondefecation days group exhibited a lower proportion of STEMI cases and a higher proportion of NSTEMI cases. Within the current study population, patients with a previous history of myocardial infarction demonstrated a significantly higher incidence of NSTEMI compared with those without (69.4% vs 43.4%; *P* < 0.001). Similarly, patients undergoing chronic hemodialysis had a significantly higher incidence of NSTEMI than those who did not (80.6% vs 44.6%; *P* < 0.001), suggesting that the differential frequencies of STEMI and NSTEMI in the 2 groups stratified by the “% of nondefecation days” can be attributed, at least in part, to these factors. Notably, the association between frequent nondefecation days and unfavorable cardiovascular prognosis remained consistent in the subgroup analyses stratified by STEMI/NSTEMI, prior history of myocardial infarction, and chronic hemodialysis.

In this study, the association between abnormal defecation patterns and cardiovascular events remained independent of various confounders. Consequently, although acute stress, impaired cardiac function, and renal dysfunction may contribute to defecation frequency, it is suggested that the abnormal defecation pattern may also be linked to cardiovascular events through distinct mechanisms. In patients with severe ACS, characterized by cardiac dysfunction, renal impairment, heart failure, and those requiring intensive treatments, such as mechanical circulatory support, a reduction in defecation frequency was not observed; rather, a higher “maximum daily defecations frequency” was noted. This observation may be associated with the higher incidence of early events observed in the high maximum daily defecation frequency group in the present study. Thus, the 2 indicators used to evaluate the defecation frequency may reflect different aspects of patient condition. There was a small group of patients (n = 56; 3.1%) whose overall hospitalization period had a low number of days with defecation, but who exhibited a high maximum daily defecation frequency on specific days. The patients in this group had the worst prognoses, which cannot be detected by the “average daily defecation frequency.”

Although the relationship between abnormal defecation patterns and prognosis may be confounded by other factors, we speculate potential mechanisms that could underlie a causal association. A long gut transit time has been reported to be associated with gut microbial diversity, composition, and metabolism, leading to chronic inflammatory responses that may elevate the risk of cardiovascular diseases.^12^, ^13^, ^14^, ^15^, ^16^ Conversely, certain types of gut microbial dysbiosis can dampen gut motility, establishing a bidirectional relationship between the gut microbiota and gut transit time.^17^ Moreover, other types of defecation pattern abnormalities, such as irritable bowel syndrome, also influence nutrient absorption and gut microbiota.^18^ In this study, stimulant laxative use was associated with high “maximum daily defecation frequency” and was also an independent predictor of poor cardiovascular outcomes. Nonetheless, the association between cardiovascular events and defecation frequency remains under investigation, and the precise mechanisms and causal relationships remain unclear. Additionally, this study was retrospective in nature, with a focus solely on defecation frequency for assessment and analysis. Precise evaluations of the gut transit time have not been performed. Nevertheless, the study was simple and noninvasive, allowing evaluation in a wide range of individuals. In contrast to previous studies that relied on questionnaire surveys, the data on defecation frequency were meticulously recorded by nurses during hospitalization, ensuring high reliability. Our findings may reflect an aspect within the broader pathological concept of impaired gut function, “gut frailty.” However, the evaluation of gut function was not conducted precisely, and variables, such as gut microbiota, metabolites originating from the gut microbiota, leaky gut syndrome, and incretin secretion, were not assessed. In fact, emerging evidence suggests that “gut frailty” may contribute to cardiovascular pathophysiology through multiple biological pathways. These include the following: 1) gut microbiome dysbiosis and altered production of gut-derived metabolites such as trimethylamine N-oxide and short-chain fatty acids;^17^^,^^19^^,^^20^ 2) increased intestinal permeability (leaky gut syndrome) and systemic low-grade inflammation;^21^ 3) autonomic imbalance with sympathetic overactivation;^22^ 4) impaired nutrient absorption; 5) reduced drug bioavailability and altered pharmacokinetics;^23^ and 6) dysregulation of gut hormone secretion including glucagon-like peptide-1 and ghrelin.^24^ These pathways provide a mechanistic framework linking abnormal defecation patterns and impaired gut function to cardiovascular risk, further supporting the concept of gut frailty as a residual risk factor in cardiovascular disease. In addition, stimulant laxative use itself may also contribute to cardiovascular risk. Beyond reflecting underlying bowel dysfunction or frailty, chronic stimulant laxative use has been reported to cause fluid and electrolyte disturbances (eg, dehydration and hypokalemia), increase sympathetic activation, and induce hemodynamic instability, all of which may exacerbate cardiovascular stress. Furthermore, long-term daily use of stimulant laxatives can lead to structural and functional impairment of the enteric nervous system, resulting in progressive colonic atony (“atonic constipation”) and reduced responsiveness to laxatives. This vicious cycle may worsen bowel dysfunction and frailty, thereby further increasing cardiovascular vulnerability. Accordingly, recent gastroenterology guidelines recommend cautious use of stimulant laxatives, particularly for long-term management.^25^^,^^26^ It should be noted, however, that our study did not include data on stimulant laxative use during the chronic phase after hospital discharge, and, therefore, the long-term impact of stimulant laxative use on cardiovascular outcomes could not be evaluated. To consider new interventions targeting the gut as residual risk, it is first necessary to elucidate the detailed mechanisms underlying the present findings. This study focused on ACS patients; however, the importance of gut function and the gut microbiome has been reported not only in atherosclerotic cardiovascular diseases but also in conditions such as left ventricular dysfunction^27^ and hypertensive heart failure.^28^ Therefore, defecation patterns may have significant implications for prognosis in other cardiovascular disease populations as well. Interventions aimed at improving gut health—including dietary fiber intake, appropriate use of laxatives, modulation of the gut microbiome, or exercise therapy—could potentially mitigate cardiovascular risk associated with gut frailty. Further investigations are necessary to determine whether interventions aimed at improving defecation patterns, such as increased dietary fiber intake, judicious use of laxatives, or adoption of moderate exercise routines, can enhance cardiovascular prognosis.

### Study Limitations

This is a retrospective, observational study; thus, it has inherent limitations, including a relatively small sample size and the inability to establish causality. There were substantial amounts of missing data. Because this was a retrospective study, we included the maximum number of patients available from the 2 participating facilities. Consequently, we did not perform a formal sample size calculation. The use of complete case analysis represents a potential limitation; however, missing data were sparse and largely nonoverlapping across covariates, mitigating the risk of substantial bias. Moreover, although extensive multivariable adjustment was performed, including up to 18 covariates in fully adjusted models, unmeasured or residual confounders that may influence defecation patterns and clinical outcomes cannot be completely excluded. Overfitting cannot be entirely excluded in analyses of outcomes with limited event numbers. Subgroup and interaction analyses were prespecified based on clinical relevance; however, these analyses should be interpreted cautiously given the potential for multiple comparisons. Given the absence of data on defecation frequency before and after hospitalization, it remains uncertain whether the observed indicators of defecation frequency are solely acute-phase phenomena or chronic conditions. Assessments of stool characteristics, including stool consistency (eg, Bristol Stool Form Scale), were not conducted. Furthermore, this study included only Japanese patients with ACS. Differences in cultural background, dietary habits, healthcare system structure, and hospitalization practices may influence bowel habits and cardiovascular outcomes. In particular, the median length of hospital stay in this cohort was 14 days, which is standard in Japan but considerably longer than in many Western countries, where early discharge is common. Therefore, these contextual factors should be considered when interpreting the results, and the findings may not be directly generalizable to other populations or healthcare settings. In this study, patients who died during hospitalization or underwent CABG were excluded before analysis. This approach may have introduced immortal time bias, as only patients who survived to the exposure assessment period were included. This potential bias should be considered when interpreting the results.

## Conclusions

Among patients discharged after hospitalization for ACS, “frequent nondefecation days” and high “maximum daily defecations frequency” during general wards stay were associated with an increased risk of future cardiovascular events. The cutoff for frequent nondefecation days (≥33.8%) corresponds to at least 1 nondefecation day every 3 days, aligning with the clinical definition of constipation. Patients with decreased defecation frequency were more likely to receive stimulant laxatives; however, the use of stimulant laxatives was independently associated with cardiovascular events. These results indicate that maintaining a consistent defecation pattern may play a crucial role in achieving better clinical outcomes. Further prospective studies are needed to confirm the findings of this study and elucidate the mechanisms underlying the relationship between defecation frequency and prognosis. Understanding these mechanisms may potentially guide clinical practice.

## Funding Support and Author Disclosures

This research was supported by a grant from the Japan Society for the Promotion of Science (JSPS) under Grant-in-Aid for Scientific Research (Kakenhi) [grant number 18K15896 and 21K08083]. The authors have reported that they have no relationships relevant to the contents of this paper to disclose.
