# Supplementary material for: Defecation Patterns and Cardiovascular Outcomes in Acute Coronary Syndrome: The Influence of Stimulant Laxative Use
Source: JACC Asia. 2026 May 16;6(7):1209–23. doi: 10.1016/j.jacasi.2026.01.035 (PMC13350099; doi:10.1016/j.jacasi.2026.01.035)
Supplement: Supplemental Figures 1-6 and Supplemental Tables 1 and 3 [file mmc1.docx]

**Supplemental Online Content**

**Title: Association of Defecation Instability and Stimulant Laxative Use with Cardiovascular Events in Acute Coronary Syndrome**

**Supplemental Figure 1.** Measurement of four indicators of defecation frequency

**Supplemental Figure 2.** Missing data pattern across variables included in the multivariable analyses.

**Supplemental Figure 3.** Distribution of defecation frequency

**Supplemental Figure 4.** Schoenfeld residual plots for defecation frequency indicators.

**Supplemental Figure 5.** Impact of a high % of non-defecation days on the primary outcome in subgroup analyses

**Supplemental Figure 6.** Impact of high maximum daily defecation frequency on primary outcome in subgroup analyses

**Supplemental Table 1.** Summary of missing data for variables included in the multivariable analyses.

**Supplemental Table 2.** Baseline patient characteristics according to frequency of non-defecation days

**Supplemental Table 3.** Baseline characteristics of patients according to maximum daily defecation frequency

**Supplemental Figure 1.** Measurement of four indicators of defecation frequency


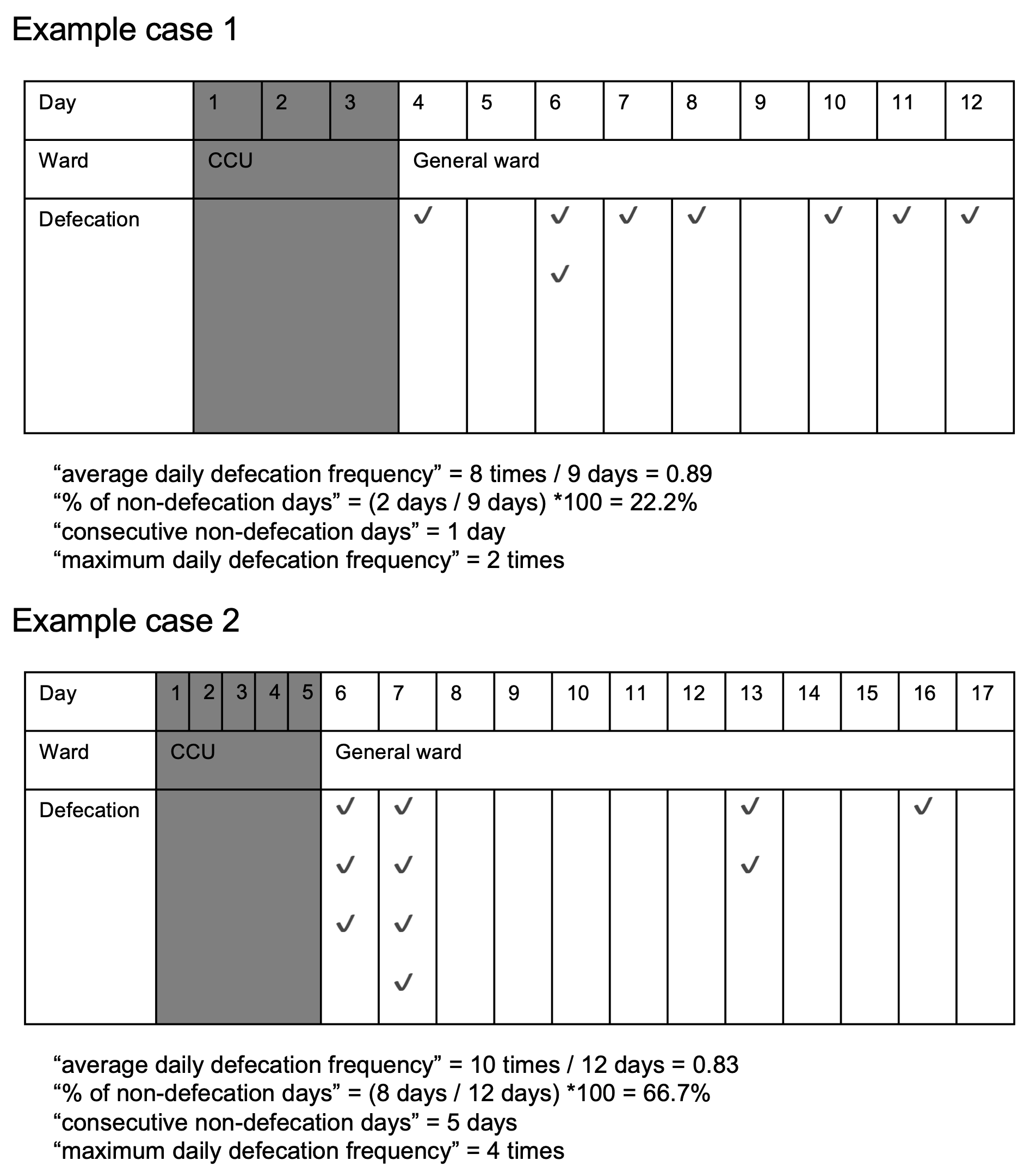


**Supplemental Figure 2.** Missing data pattern across variables included in the multivariable analyses.

**
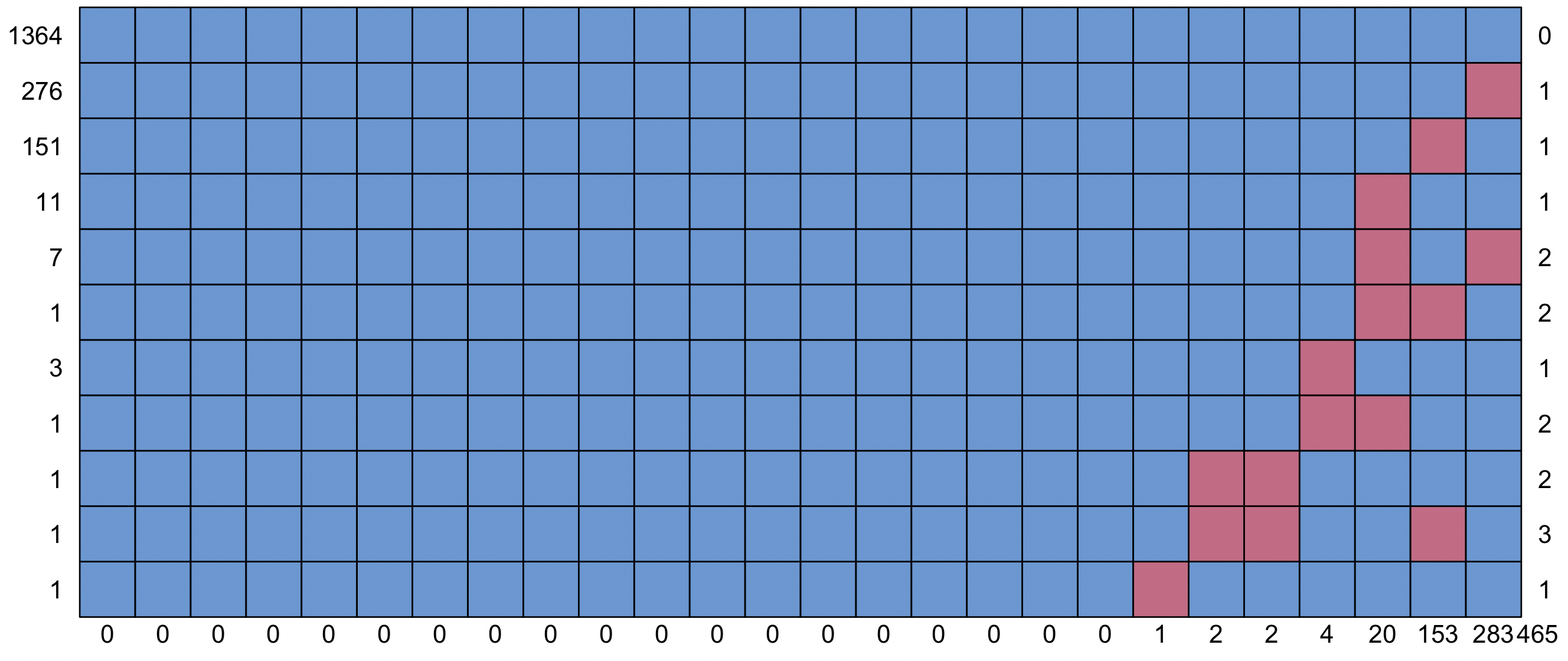
**

Blue cells indicate observed values and red cells indicate missing values.

Missingness occurred predominantly in isolated variables without systematic overlap across covariates.

**Supplemental Figure 3.** Distribution of defecation frequency


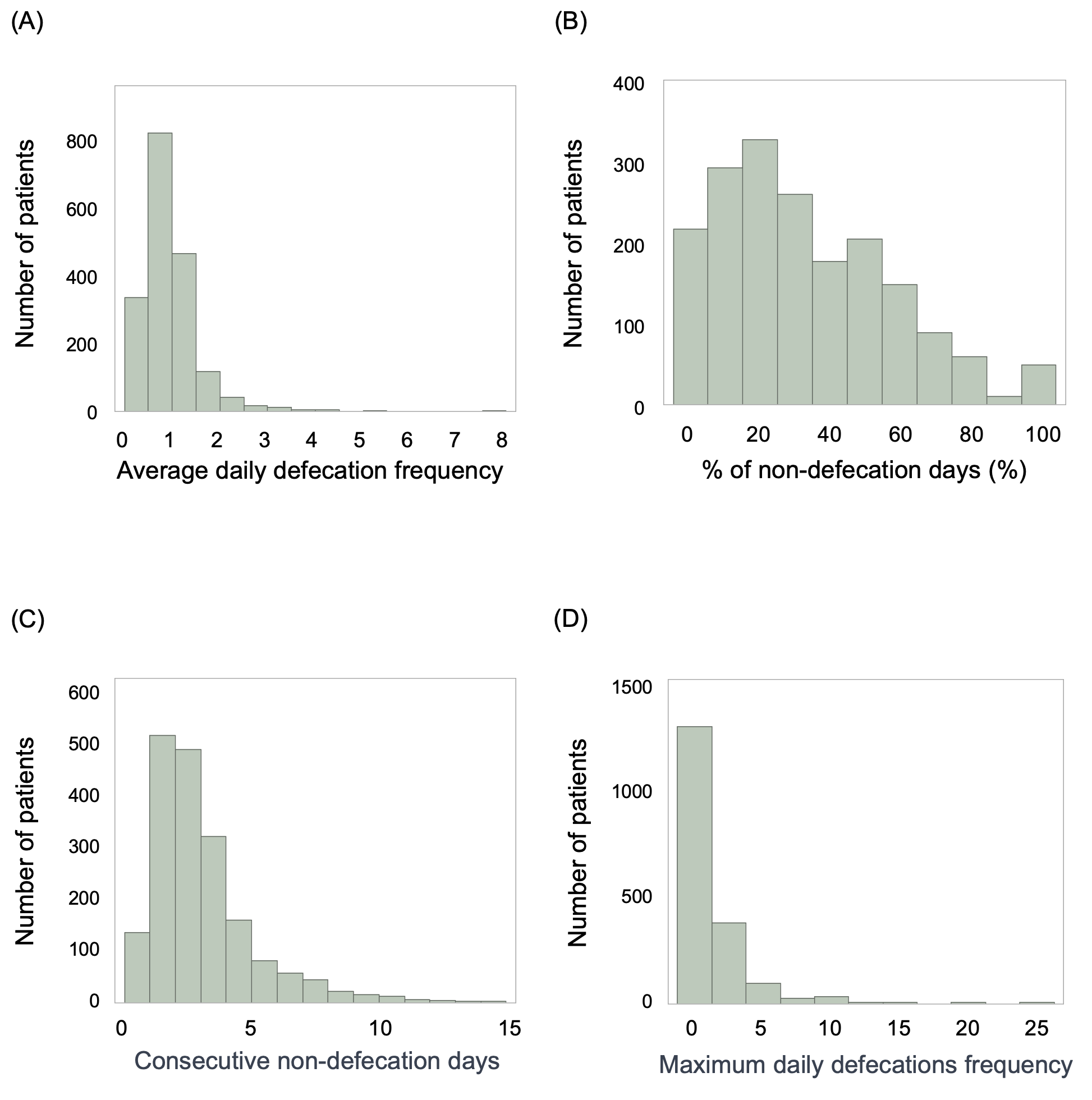


Distribution of four indicators of defecation frequency: (A) “average daily defecation frequency,” (B) “frequency of non-defecation days,” (C) “consecutive non-defecation days,” and (D) “maximum daily defecation frequency.”

**Supplemental Figure 4.** Schoenfeld residual plots for defecation frequency indicators.

**
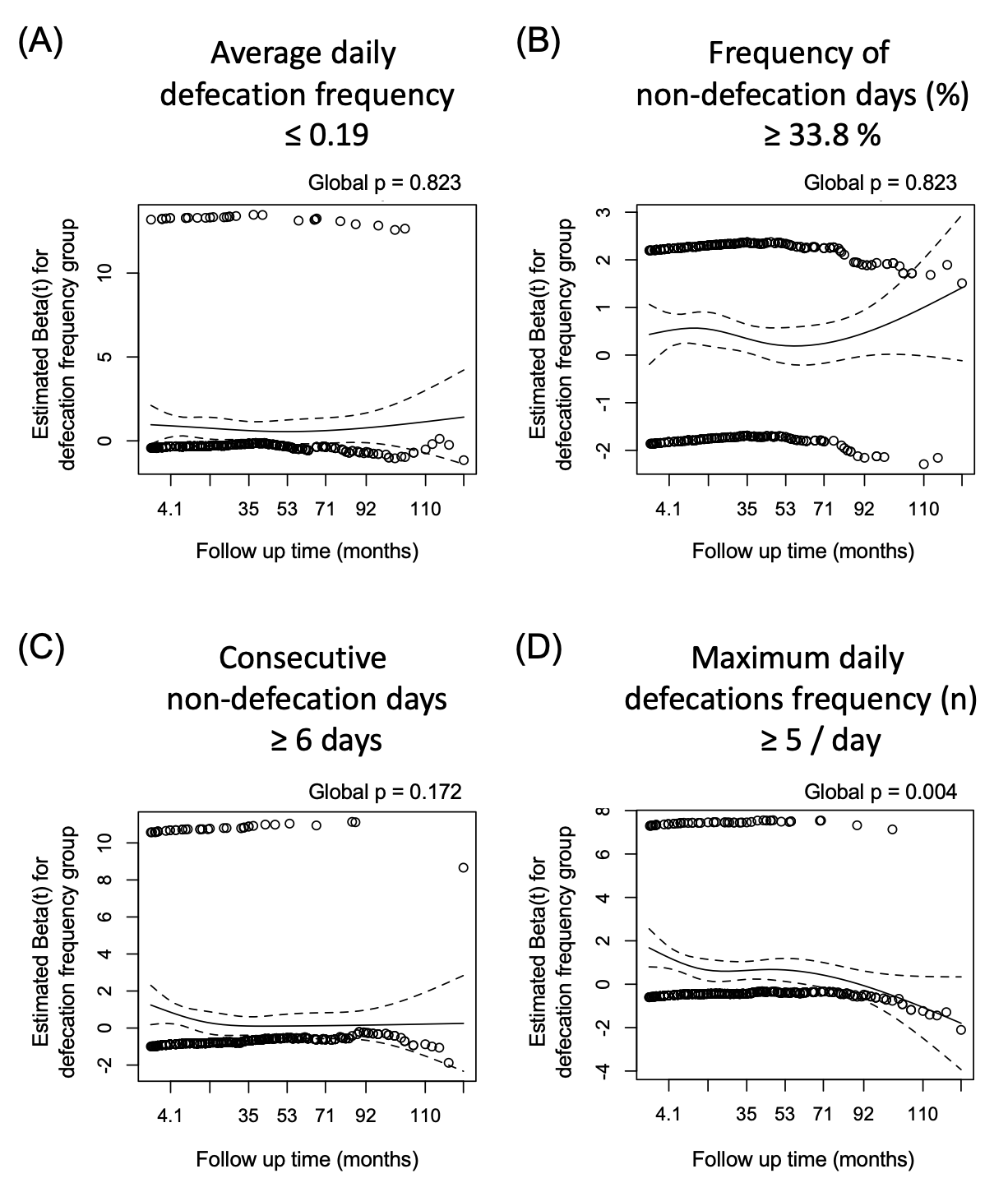
**

Schoenfeld residual plots for the four defecation frequency indicators. The y-axis represents the estimated time-varying regression coefficient β(t), corresponding to the log hazard ratio, derived from Schoenfeld residuals. Dashed lines indicate 95% confidence intervals. Global p-values are shown for tests of the proportional hazards assumption.

**Supplemental Figure 5.** Impact of a hifh % of non-defecation days on the primary outcome in subgroup analyses


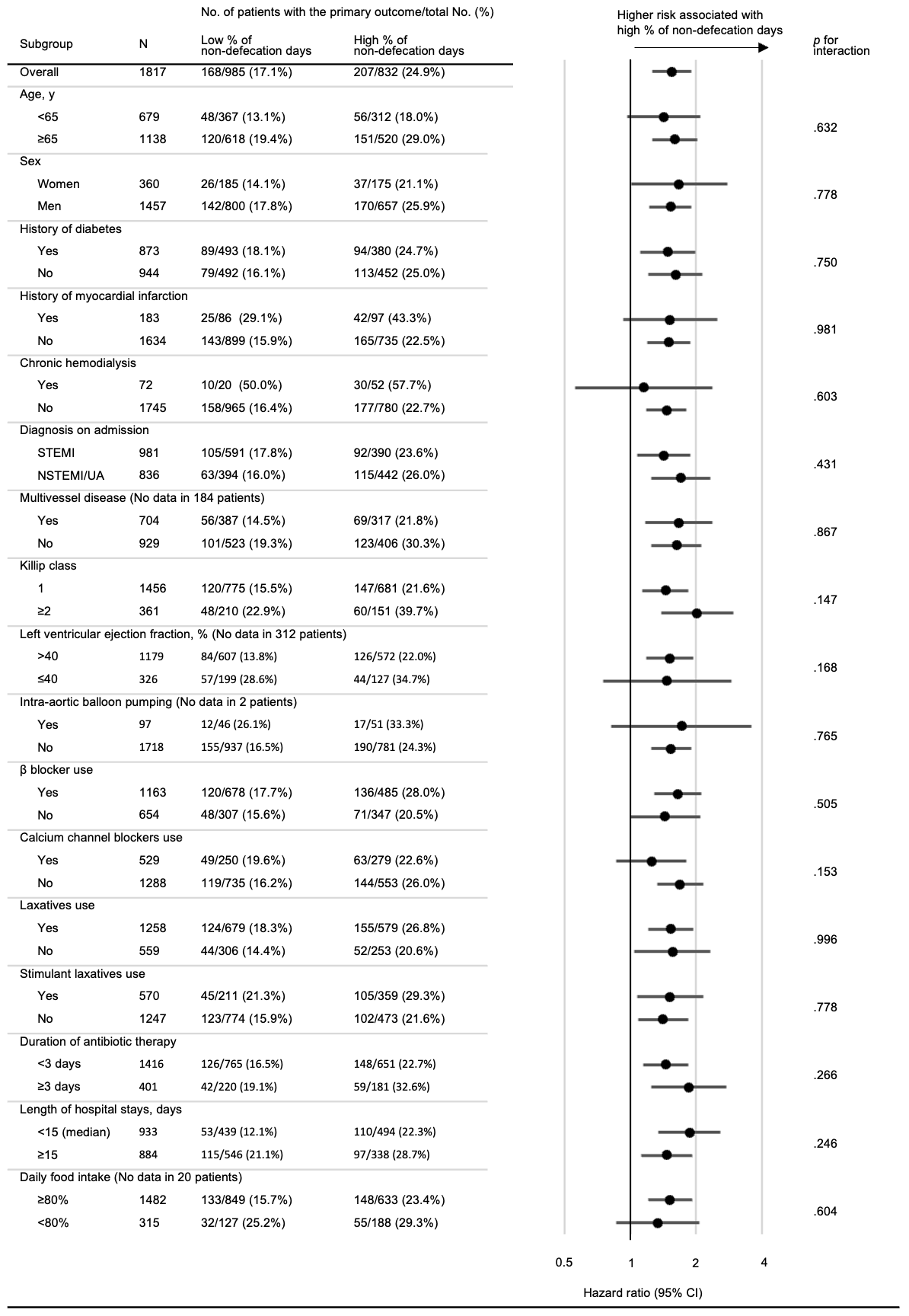


**Supplemental Figure 6.** Impact of high maximum daily defecation frequency on the primary outcome in subgroup analyses


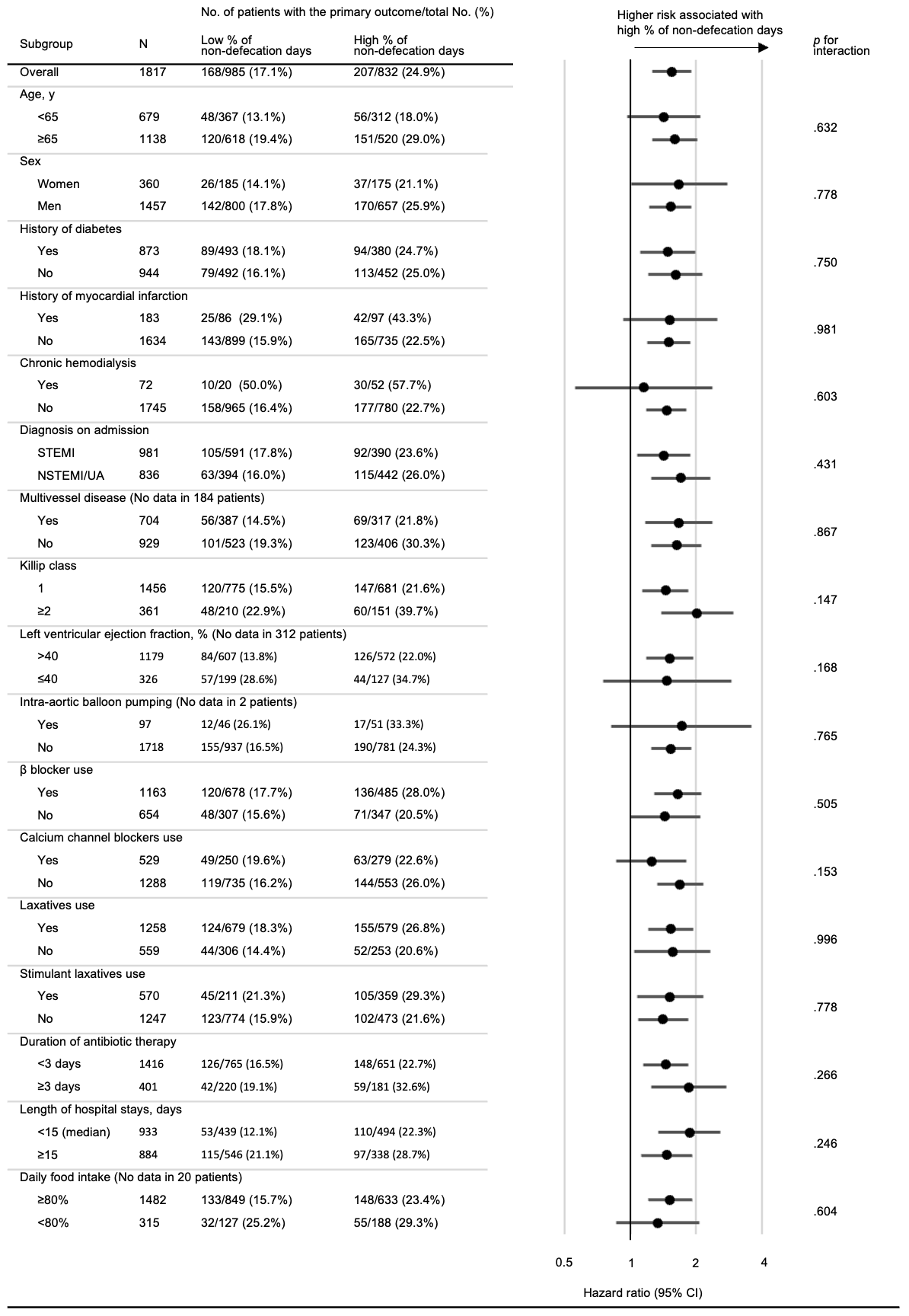


| **Supplemental Table 1.** Summary of missing data for variables included in the multivariable analyses. | |
| --- | --- |
| Variables | Missing number (%) |
| Age | 0 (0%) |
| Male sex | 0 (0%) |
| Hypertension | 1 (0.1%) |
| Diabetes mellitus | 0 (0%) |
| Dyslipidemia | 0 (0%) |
| Current smoker | 4 (0.2%) |
| Prior myocardial infarction | 0 (0%) |
| eGFR | 283 (15.6%) |
| STEMI | 0 (0%) |
| Killip class | 0 (0%) |
| Percutaneous coronary intervention | 153 (8.4%) |
| Intra-aortic balloon pumping | 2 (0.1%) |
| VA-ECMO | 2 (0.1%) |
| Daily food intake | 20 (1.1%) |
| Length of hospital stays | 0 (0%) |
| Laxatives | 0 (0%) |
| Intestinal regulators | 0 (0%) |
| Antibiotic use | 0 (0%) |

Data are shown as numbers (%).

eGFR, estimated glomerular filtration rate; STEMI, ST-elevation acute myocardial infarction; VA-ECMO, venoarterial extracorporeal membrane oxygenation.

| **Supplemental Table 2.** Baseline characteristics of the patients according to the % of non-defecation days | | | |
| --- | --- | --- | --- |
|  | Frequency of non-defecation days | |  |
|  | Low  <33.8％  n=985 | High  ≥33.8％  n=832 | *p* value |
| Age, years | 67.4±12.6 | 67.7±12.4 | .530 |
| Male sex | 81.2% | 79.0% | .231 |
| Body mass index, kg/m^2^ | 24.3±3.8 | 24.2±3.9 | .507 |
| Hypertension | 56.2% | 54.8% | .552 |
| Diabetes mellitus | 50.1% | 45.7% | .063 |
| Dyslipidemia | 61.5% | 63.9% | .288 |
| Current smoker | 26.7% | 28.9% | .283 |
| Prior myocardial infarction | 8.7% | 11.7% | .039 |
| eGFR, mL/min/1.73m^2^ | 63.7±21.8 | 63.3±23.6 | .726 |
| Chronic hemodialysis | 2.0% | 6.3% | <.001 |
| Diagnosis on admission |  |  |  |
| STEMI | 60.0% | 46.9% | <.001 |
| Multivessel disease | 42.5% | 43.9% | .593 |
| Killip class ≥2 | 21.3% | 18.2% | .092 |
| LVEF on admission, % | 52.1±13.0 | 54.5±12.9 | <.001 |
| Percutaneous coronary intervention | 92.7% | 89.6% | .026 |
| Intra-aortic balloon pumping | 4.7% | 6.1% | .171 |
| VA-ECMO | 0.6% | 1.3% | .117 |
| Length of hospital stays, days | 15 (11–21) | 13 (9–18) | <.001 |
| Length of a general ward stays, days | 13 (9–17) | 11 (7–16) | <.001 |
| Daily food intake ≥ 80% | 87.0% | 77.1% | <.001 |
| Antibiotic use | 78.4% | 80.5% | .259 |
| Duration of antibiotic therapy, days | 1 (1–2) | 1 (1–2) | .986 |
| Antibiotic therapy ≥3 days | 22.3% | 21.8% | .766 |
| Medications on discharge |  |  |  |
| β-blockers | 68.8% | 58.3% | <.001 |
| Calcium channel blockers | 25.4% | 33.5% | <.001 |
| Laxatives | 68.9% | 69.6% | .762 |
| Stimulant laxatives | 21.4% | 43.2% | <.001 |
| Intestinal regulators | 2.8% | 4.3% | .088 |
| NSAIDs | 1.2% | 1.0% | .600 |
| Maximum daily defecations frequency | 2 (2-3) | 1 (1-2) | <.001 |
| Maximum daily defecations frequency ≥5 | 10.8% | 6.7% | .003 |

Data are shown as means±standard deviations, medians (interquartile ranges), or numbers. (%).

eGFR, estimated glomerular filtration rate; LVEF, left ventricular ejection fraction; NSAIDs, nonsteroidal anti-inflammatory drugs; STEMI, ST-elevation acute myocardial infarction; VA-ECMO, venoarterial extracorporeal membrane oxygenation.

| **Supplemental Table 3.** Baseline characteristics of the patients according to the maximum daily defecation frequency | | | |
| --- | --- | --- | --- |
|  | Maximum daily defecations frequency | |  |
|  | Low  ≤4  n=1655 | High  ≥5  n=162 | *p* value |
| Age, years | 67.3±12.5 | 69.7±11.3 | .021 |
| Male sex | 80.0% | 82.1% | .522 |
| Body mass index, kg/m^2^ | 24.3±3.8 | 23.6±4.2 | .054 |
| Hypertension | 55.7% | 54.3% | .739 |
| Diabetes mellitus | 48.2% | 46.9% | .762 |
| Dyslipidemia | 63.1% | 57.4% | .150 |
| Current smoker | 27.7% | 27.2% | .875 |
| Prior myocardial infarction | 10.1% | 9.9% | .931 |
| eGFR, mL/min/1.73m^2^ | 64.2±22.5 | 55.9±22.5 | <.001 |
| Chronic hemodialysis | 4.0% | 3.7% | .860 |
| Diagnosis on admission |  |  |  |
| STEMI | 53.8% | 56.2% | .559 |
| Multivessel disease | 44.4% | 30.6% | <.001 |
| Killip class ≥2 | 17.5% | 44.4% | <.001 |
| LVEF on admission, % | 53.6±12.8 | 48.8±14.1 | <.001 |
| Percutaneous coronary intervention | 91.2% | 92.4% | .619 |
| Intra-aortic balloon pumping | 4.3% | 16.1% | <.001 |
| VA-ECMO | 0.7% | 3.1% | .003 |
| Length of hospital stays, days | 14 (9–18) | 25.5 (16–43) | <.001 |
| Length of a general ward stays, days | 12 (8–16) | 21 (14–35) | <.001 |
| Daily food intake ≥80% | 84.0% | 66.5% | <.001 |
| Antibiotic use | 78.7% | 86.4% | .020 |
| Duration of antibiotic therapy, days | 1 (1–2) | 3 (1–10) | <.001 |
| Antibiotic therapy ≥3 days | 18.9% | 54.3% | <.001 |
| Medications on discharge |  |  |  |
| β-blockers | 63.1% | 72.8% | .012 |
| Calcium channel blockers | 30.0% | 19.8% | .004 |
| Laxatives | 68.5% | 76.5% | .031 |
| Stimulant laxatives | 29.7% | 48.8% | <.001 |
| Intestinal regulators | 2.3% | 16.1% | <.001 |
| NSAIDs | 1.2% | 0.6% | .537 |
| % of non-defecation days | 33.3 (18.2-53.8) | 25.3 (12.5-38.1) | <.001 |
| % of non-defecation days ≥33.8% | 46.9% | 34.6% | .003 |

Data are shown as means±standard deviations, medians (interquartile ranges), or numbers. (%).

eGFR, estimated glomerular filtration rate; LVEF, left ventricular ejection fraction; NSAIDs, nonsteroidal anti-inflammatory drugs; STEMI, ST-elevation acute myocardial infarction; VA-ECMO, venoarterial extracorporeal membrane oxygenation.
